# Responses of Male and Female Cucumber Flowers to *CsHXK2*-Mediated Sugar Metabolism

**DOI:** 10.3390/plants15182791

**Published:** 2026-09-11

**Authors:** Dongqin Zhang, Dong Hou, Panpan Duan, Huiting Wang, Hongzhong Yue, Yali Li

**Affiliations:** Vegetable Institute, Gansu Academy of Agricultural Sciences, Lanzhou 730070, China; zhangdongqin@gsagr.cn (D.Z.); panpanduan1002@163.com (P.D.); 15101453633@163.com (H.W.); tianyuan2828@163.com (H.Y.); yaligsau@163.com (Y.L.)

**Keywords:** *CsHXK2*, cucumber male/female floral organs, sugar metabolism and signal

## Abstract

Plant hexokinase (HXK) is a key enzyme in carbohydrate metabolism that serves a dual function by catalyzing hexose phosphorylation and participating in sugar signaling. In this study, expression pattern analysis and reverse-genetic approaches were used to investigate the role of *CsHXK2* in cucumber floral development. *CsHXK2* was predominantly expressed in the stamens and nectary of male flowers and in the stigma, nectary, and ovary of female flowers. The CsHXK2 protein was localized to chloroplasts. Silencing *CsHXK2* significantly increased glucose, fructose, and sucrose levels in the examined tissues of both male and female flowers. This increase was accompanied by reduced activities of HXK, PFK, AI, SS-I, and NI and enhanced activities of SPS and SS-II, with these changes being more pronounced in female flowers than in male flowers. Gene expression analysis further revealed that *CsHXK2* silencing induced tissue-specific changes in the expression of sugar-metabolism-related genes in both male and female flowers. These results demonstrate that *CsHXK2* contributes to the maintenance of sugar homeostasis in cucumber floral organs. Moreover, male and female floral organs exhibit distinct responses to HXK-mediated regulation of sugar metabolism, suggesting that *CsHXK2* may influence cucumber flower development by modulating carbon allocation and sugar signaling.

## 1. Introduction

Sucrose is the primary carbohydrate produced through photosynthesis in plants and is transported via the phloem from source tissues to non-photosynthetic sink tissues [1]. In sink tissues, sucrose can either be stored or metabolized into hexoses such as glucose and fructose. Following phosphorylation, these hexoses enter downstream metabolic pathways [2]. In plants, hexokinase (HXK) phosphorylates glucose to glucose-6-phosphate, thereby initiating glycolysis and contributing to the production of energy and key metabolic intermediates required for plant growth and development [3]. HXK has dual functions in plants: in addition to its catalytic role in glucose phosphorylation, it acts as a glucose sensor involved in the regulation of gene expression, hormonal interactions, and plant growth and development. Crystallographic studies of the rice hexokinase *OsHXK7* have shown that glucose binding induces conformational changes, with Gly_76_ and Trp_256_ identified as key residues involved in maintaining its catalytic activity and fluorescence properties [4]. Such glucose-induced conformational changes may not only influence HXK catalytic activity but also contribute to its function in signal transduction. In *Arabidopsis* pollen, HXK, an important component of sugar metabolism and signaling, exhibits reduced sensitivity to fructose [5]. Beyond its enzymatic function, HXK also acts as an important sugar sensor that perceives changes in cellular sugar status and regulates downstream physiological responses [6].

Based on the N-terminal amino acid sequence, plant *HXKs* are generally classified into four types: A, B, C, and D [7]. Type A HXKs, including *AtHXK3*, *NtHXK2*, *LeHXK4*, and *OsHXK4*, possess an N-terminal chloroplast transit peptide that facilitates their targeting to plastids [8,9]. Type B HXKs typically contain an N-terminal hydrophobic membrane-anchoring domain associated with mitochondrial targeting. Members of this group include *AtHXK1/2*, *AtHKL1/2/3*, *OsHXK2/3/5/6/9/10* [9], *LeHXK1-3*, and *ZmHXK3a/3b/4/5/6/9/10* [10]. Certain Type B HXKs can also localize to the nucleus, possibly owing to nuclear localization signals located near their transmembrane domains [11]. Type C HXKs, such as *OsHXK1/7/9* have been identified exclusively in monocotyledons and the moss *Physcomitrella patens*. These *HXKs* lack both a membrane-anchoring domain and a chloroplast-targeting peptide and are localized to the nucleus and cytoplasm [12,13]. In contrast, Type D HXKs have been identified exclusively in gymnosperms, lycophytes, and bryophytes, and members of this group may lack conserved peptide motifs [13].

To date, the functions of plant *HXK* genes have been characterized in numerous species, particularly in relation to abiotic stress responses and the regulation of plant growth and development. For instance, the apple hexokinase *MdHXK1* phosphorylates the basic helix-loop-helix (bHLH) transcription factor *MdbHLH3*, thereby promoting anthocyanin biosynthesis [14]. Under low-temperature conditions, reduced HXK activity in apple buds is associated with decreased levels of glucose-6-phosphate (G6P) and glutathione [15], whereas *CsHXK3* expression in tea plants is markedly upregulated in response to cold stress [16]. The B-type hexokinase *GmHXK15* is specifically targeted to mitochondria and exhibits HXK catalytic activity. Overexpression of *GmHXK15* significantly enhances alkaline stress tolerance in transgenic soybean plants [10]. In tea plants (*Camellia sinensis*), the B-type hexokinase *CsHXK4* exhibits catalytic activity and is localized to both the nucleus and mitochondria [3]. Overexpression of *CsHXK4* from tea plant in *Arabidopsis thaliana* induces glucose hypersensitivity, enhances photosynthetic capacity, increases sugar accumulation, and contributes to the maintenance of cell membrane stability. It also upregulates key genes in the CBF signaling pathway [3]. Overexpression of *AtHXK1* in tobacco reduces stomatal conductance and transpiration without adversely affecting plant growth, thereby enhancing tolerance to drought and salt stress [17]. The *HXK1* signaling pathway is also involved in regulating shoot branching and interacts synergistically with indole-3-acetic acid (IAA), jasmonic acid, and cytokinins to modulate plant architecture [18].

Cucumber (*Cucumis sativus* L.), one of the most widely cultivated vegetable crops, has high commercial value and broad consumer acceptance worldwide [19]. Understanding the regulatory mechanisms underlying cucumber quality is essential for improving breeding efficiency and developing high-quality horticultural products. *HXK* genes have been identified and functionally characterized in numerous plant species, demonstrating their important roles in plant metabolism, sugar signaling, and responses to biotic and abiotic stresses. However, the biological function of *CsHXK2* in cucumber remains largely unknown. Therefore, elucidating the role of *CsHXK2* may provide insights into its biological functions and offer a theoretical basis for cucumber genetic improvement. Our previous study identified six *CsHXK* genes, among which *CsHXK2* (*CsaV3_1G039830.1*), a homolog of *AtHXK2*, exhibited relatively high expression in floral tissues. Based on these findings, quantitative real-time PCR (qRT-PCR) was used to analyze the expression patterns of *CsHXK2* in different cucumber tissues and in the floral organs of the female line C50 and the male line m38. In addition, a transient expression system in tobacco was used to determine the subcellular localization of the *CsHXK2* protein. Finally, virus-induced gene silencing (VIGS) was employed to silence *CsHXK2*, and the resulting phenotypes, sugar contents, activities of key sugar-metabolic enzymes, and expression levels of sugar metabolism-related genes were subsequently analyzed. This study aimed to elucidate the role of *CsHXK2* in cucumber flower development and to investigate its involvement in the regulation of sugar metabolism in male and female floral organs. The findings are expected to provide insights into the physiological and molecular mechanisms associated with floral organ development and reproductive abnormalities, and to provide a theoretical basis and potential candidate targets for improving protected cucumber cultivation and molecular breeding.

## 2. Results

### 2.1. Expression Analysis and Subcellular Localization of CsHXK2

**Figure 1:**
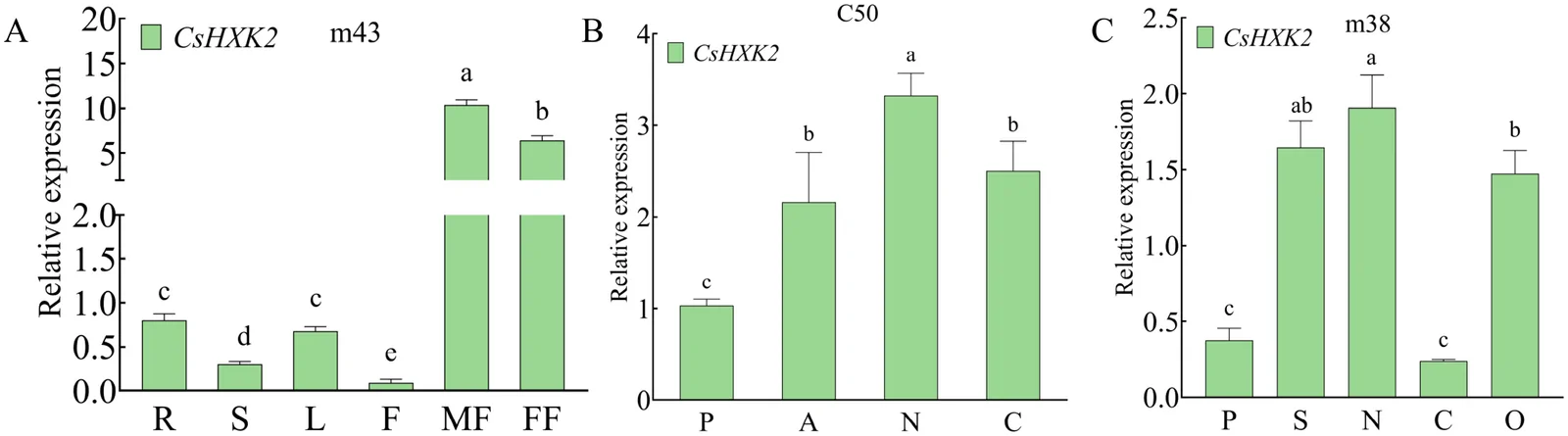
Issue-specific expression patterns of *CsHXK2* in m43 (**A**); Expression levels of *CsHXK2* in different floral tissues of male flowers in C50 (**B**); Expression levels of *CsHXK2* in different floral tissues of female flowers in m38 (**C**). Note: R, root; S, stem; L, leaf; F, fruit (20 DAF); MF, male flower; FF, female flower; P, petal; A, anther; N, nectary; C, calyx; Sti, stigma; O, ovary. The same abbreviations are used throughout the manuscript. Different lowercase letters indicate significant differences among treatments (*p* < 0.05), whereas the same letter indicates no significant difference.

*CsHXK2* was expressed in various tissues of m43, with significantly higher expression levels in male and female flowers than in the other tissues examined. In the male line C50, *CsHXK2* was expressed in all examined floral organs, with significantly higher expression levels in the nectary, anthers, and calyx than in the petals. In the female line m38, *CsHXK2* was also expressed in all examined floral organs, with significantly higher expression levels in the nectary, stigma, and ovary than in the petals and calyx (Figure 1).

**Figure 2:**
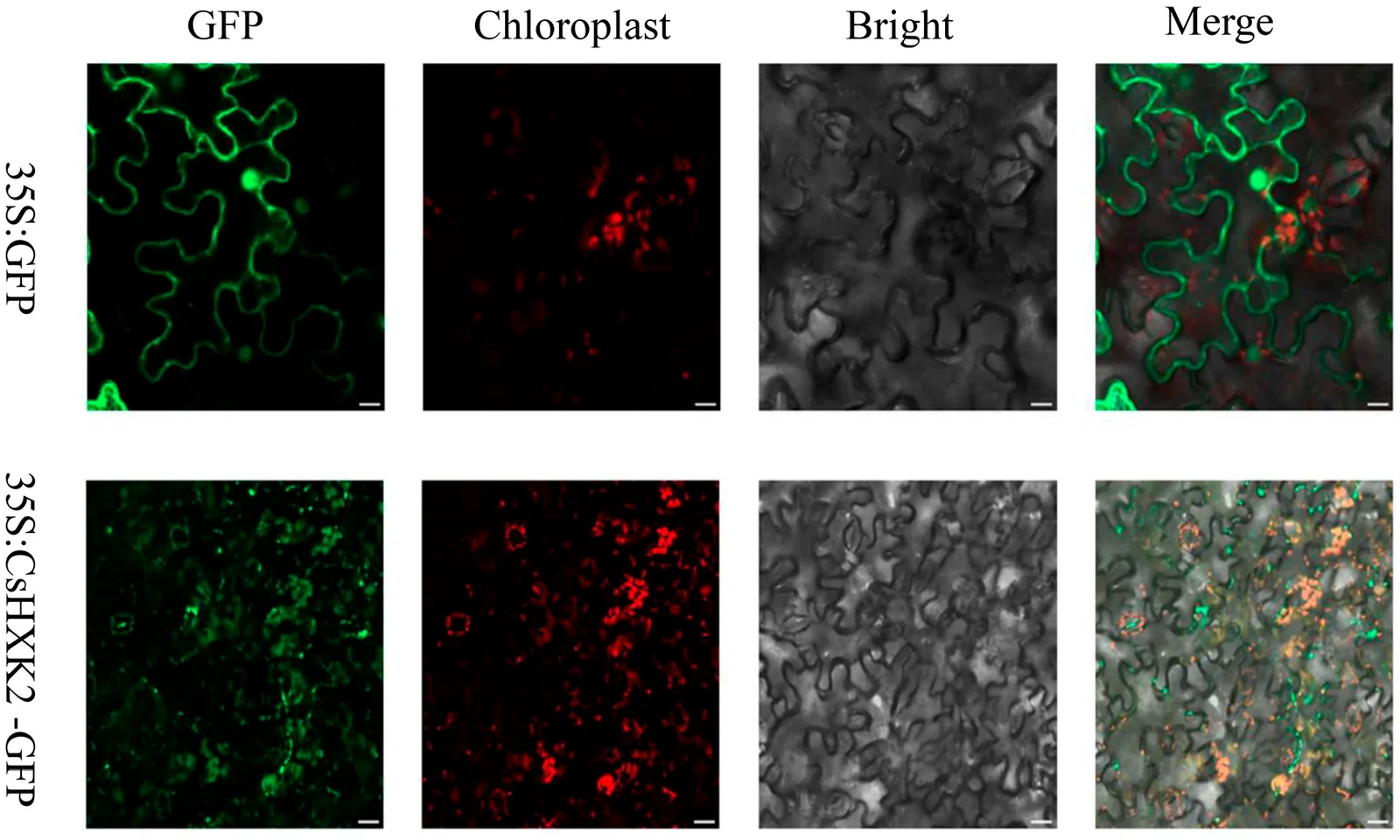
Subcellular localization of CsHXK2 in tobacco. The scale bar represents 50 μm. Note: Green represents GFP fluorescence of the *CsHXK2*-GFP fusion protein; red indicates chlorophyll autofluorescence of chloroplasts; orange is the merged signal of green GFP fluorescence and red chlorophyll autofluorescence.

As shown in Figure 2, chloroplasts exhibited red autofluorescence, whereas the CsHXK2-GFP fusion protein produced green fluorescence. In the merged image, the green GFP signal overlapped with the red chloroplast autofluorescence, producing a yellow signal. These results indicate that CsHXK2 is localized to chloroplasts.

### 2.2. Silencing Efficiency

**Figure 3:**
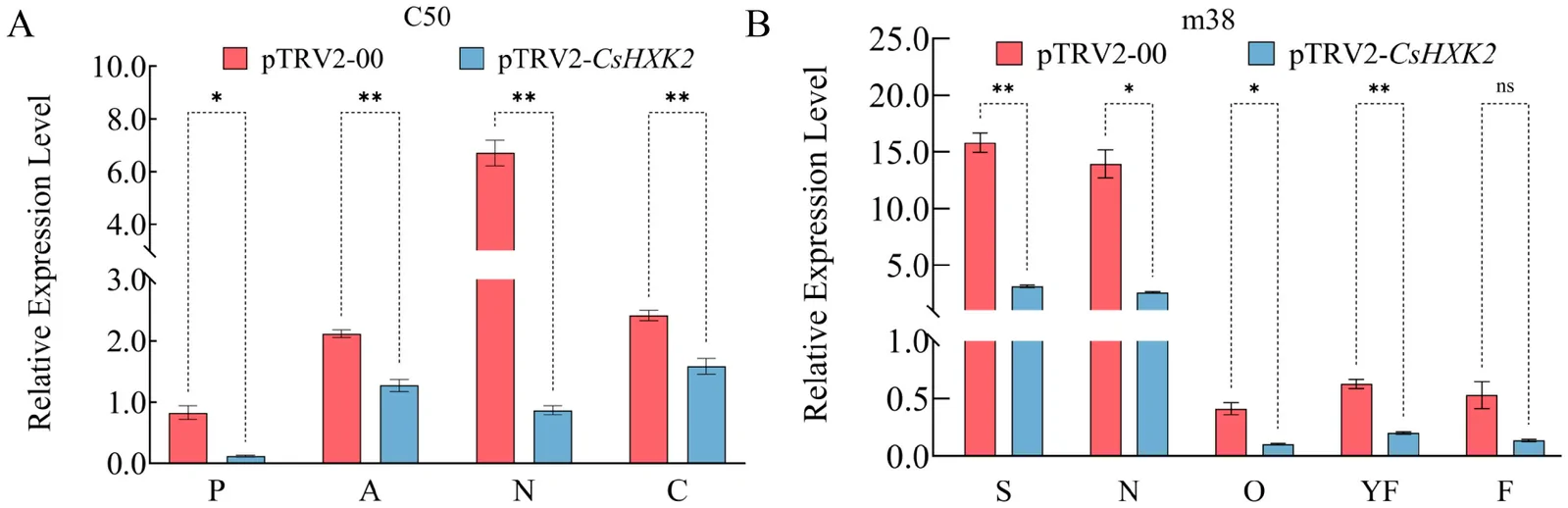
Silencing efficiency of *CsHXK2* in the male line C50 (**A**) and the female line m38 (**B**). Note: P, petal; A, anther; N, nectary; C, calyx; S, stigma; O, ovary; YF, young fruit; F, marketable fruit. Note: *, *p* < 0.05, **, *p* < 0.01, and ns, not significant (*p* > 0.05).

As shown in Figure 3, *CsHXK2* expression levels in the anthers and nectaries of the male line C50 were significantly reduced by 39.96% and 87.07%, respectively, in pTRV2-*CsHXK2* plants compared with the pTRV2-00 empty-vector control. In the female line m38, *CsHXK2* expression levels in the stigmas, nectaries, and ovaries were significantly reduced by 80.19%, 81.36%, and 74.43%, respectively, in pTRV2-*CsHXK2* plants compared with the pTRV2-00 control.

### 2.3. Soluble Sugar Contents in Male Floral Organs

**Figure 4:**
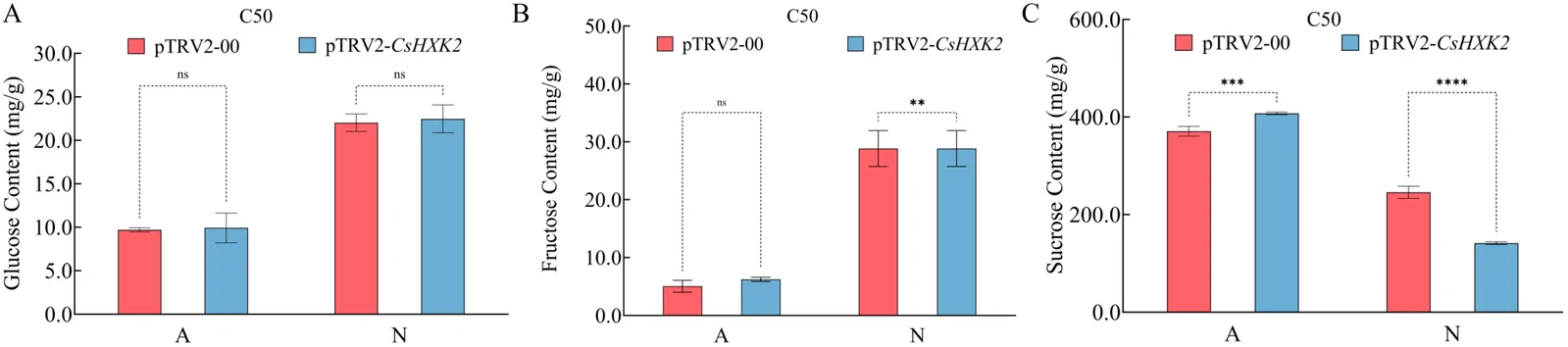
Effects of silencing on the levels of glucose (**A**), fructose (**B**) and sucrose (**C**) in C50 anthers and nectaries. Note: **, *p* < 0.01, ***, *p* < 0.001, ****, *p* < 0.0001 and ns, not significant (*p* > 0.05).

As shown in Figure 4, glucose, fructose, and sucrose contents in the anthers of *CsHXK2*-silenced C50 plants were significantly increased by 98.26%, 67.19%, and 12.21%, respectively, compared with the control. Following *CsHXK2* silencing, glucose, fructose, and sucrose contents in the nectaries of C50 plants were significantly increased by 54.99%, 43.62%, and 73.59%, respectively, compared with the control. The glucose and fructose contents were significantly higher in the nectaries than in the anthers, whereas the sucrose content was significantly lower in the nectaries than in the anthers.

### 2.4. Soluble Sugar Contents in Female Floral Organs

**Figure 5:**
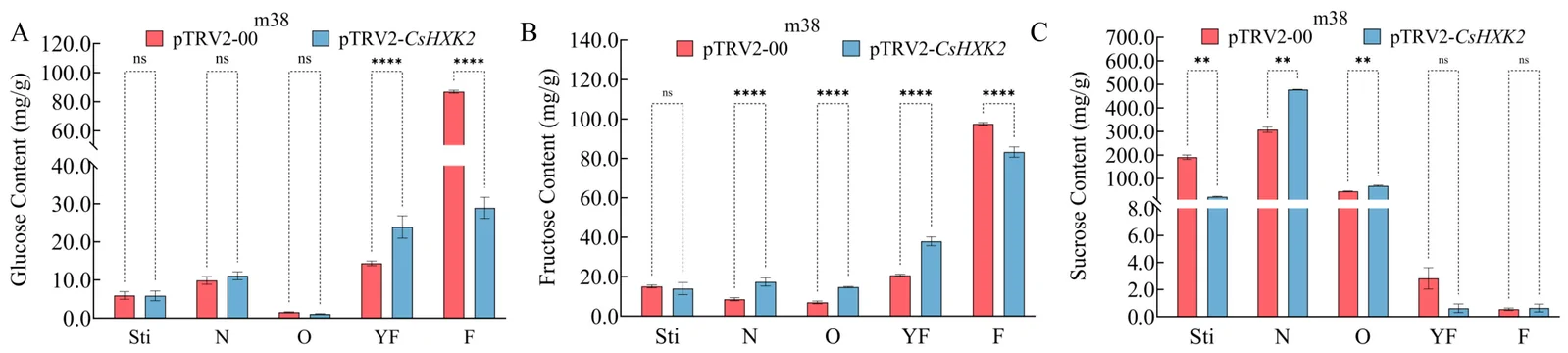
Effects of *CsHXK2* silencing on glucose (**A**), fructose (**B**), and sucrose (**C**) contents in the stigmas, nectaries, and ovaries of the female line m38. Note: **, *p* < 0.01, ****, *p* < 0.0001 and ns, not significant (*p* > 0.05).

As shown in Figure 5, glucose contents in the stigmas, nectaries, and ovaries of pTRV2-*CsHXK2* plants were significantly increased by 43.43%, 106.76%, and 314.38%, respectively, compared with the control. Fructose contents in the stigmas, nectaries, and ovaries of pTRV2-*CsHXK2* plants were significantly increased by 32.58%, 103.15%, and 121.32%, respectively, compared with the control. Similarly, sucrose contents in the stigmas, nectaries, and ovaries of pTRV2-*CsHXK2* plants were significantly increased by 398.67%, 54.07%, and 50.71%, respectively, compared with the control. Among the three floral organs examined, the nectaries exhibited the highest contents of glucose, fructose, and sucrose, followed by the stigmas, whereas the ovaries exhibited the lowest contents.

### 2.5. Sugar Metabolism-Related Enzyme Activities in Male Floral Organs

Compared with the control, the activities of HXK (hexokinase), FRK (fructokinase), NI (neutral invertase), SS-I (sucrose synthase in the cleavage direction), and AI (acid invertase) in the anthers of *CsHXK2*-silenced plants decreased by 10.31%, 25.10%, 20.15%, 5.90%, and 14.36%, respectively. In contrast, SPS (sucrose-phosphate synthase) and SS-II (sucrose synthase in the synthesis direction) activities increased by 45.89% and 14.98%, respectively.

**Figure 6:**
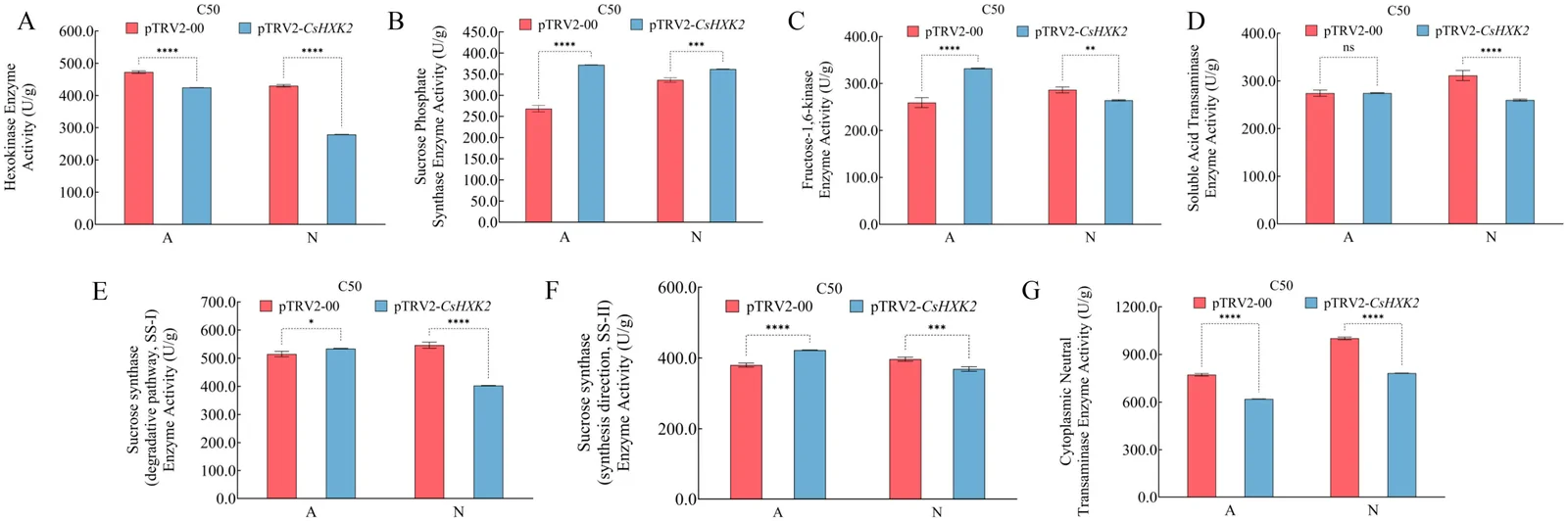
Effects of silencing on the activity of sugar-metabolizing enzymes in C50 anthers and nectaries. (**A**): Hexokinase, HXK; (**B**): Sucrose Phosphate Synthase, SPS; (**C**): Fructokinase, FRK; (**D**): Neutral invertase, NI; (**E**): Sucrose Synthase (degradative pathway, SS-I); (**F**): Sucrose Synthase (synthesis direction, SS-II); (**G**): Acid Invertase, AI. Same as below. Note: *, *p* < 0.05, **, *p* < 0.01, ***, *p* < 0.001, ****, *p* < 0.0001 and ns, not significant (*p* > 0.05).

In the nectaries of pTRV2-*CsHXK2* plants, SPS and SS-II activities increased by 4.70% and 19.27%, respectively, compared with the control. Conversely, HXK, FRK, NI, SS-I, and AI activities in the nectaries decreased by 37.62%, 22.88%, 23.42%, 26.27%, and 18.26%, respectively, compared with the control. Furthermore, SS-II activity did not differ significantly between the anthers and nectaries. However, SPS and NI activities were significantly higher in the nectaries than in the anthers, whereas HXK, FRK, SS-I, and AI activities were significantly higher in the anthers than in the nectaries (Figure 6).

### 2.6. Sugar Metabolism-Related Enzyme Activities in Female Floral Organs

**Figure 7:**
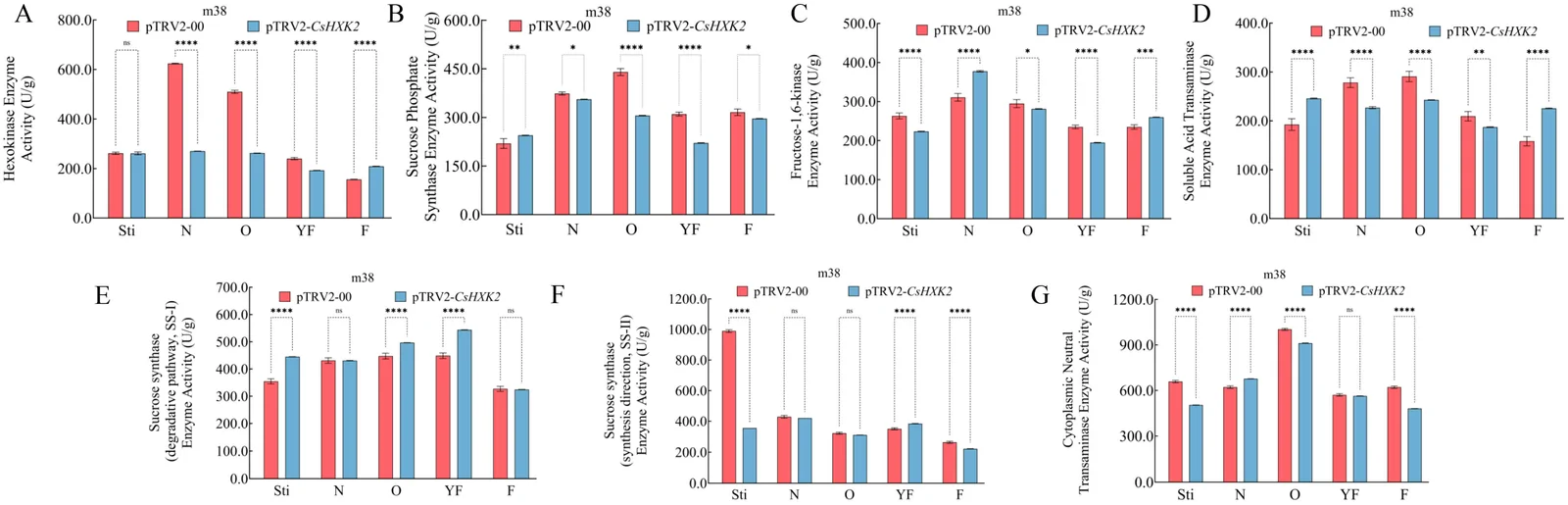
Effects of silencing on the activity of sugar-metabolizing enzymes in m38 stigma, nectary, and ovary. (**A**): HXK; (**B**): SPS; (**C**): FRK; (**D**): NI; (**E**): SS-I; (**F**): SS-II; (**G**): AI. Note: *, *p* < 0.05, **, *p* < 0.01, ***, *p* < 0.001, ****, *p* < 0.0001 and ns, not significant (*p* > 0.05).

In the stigmas of *CsHXK2*-silenced plants, SPS and SS-II activities were significantly increased by 20.55% and 159.04%, respectively, compared with the control. In contrast, HXK, FRK, NI, SS-I, and AI activities were significantly decreased by 21.57%, 15.01%, 25.67%, 20.09%, and 26.55%, respectively (Figure 7).

In the nectaries of *CsHXK2*-silenced plants, SPS and SS-II activities were significantly increased by 14.79% and 13.35%, respectively, compared with the control. Conversely, HXK, FRK, NI, SS-I, and AI activities were significantly decreased by 59.43%, 16.99%, 10.35%, 7.55%, and 24.10%, respectively.

In the ovaries of *CsHXK2*-silenced plants, SPS and SS-II activities were significantly increased by 35.77% and 23.74%, respectively, compared with the control. In contrast, HXK, FRK, NI, SS-I, and AI activities were significantly decreased by 46.23%, 20.34%, 11.15%, 14.60%, and 26.78%, respectively.

Furthermore, HXK, SPS, and FRK activities followed the order nectaries > ovaries > stigmas, whereas NI and AI activities followed the order ovaries > nectaries > stigmas. SS-I and SS-II activities followed the order ovaries > stigmas > nectaries.

### 2.7. Expression Levels of Sugar Metabolism-Related Genes in Male Floral Organs

**Figure 8:**
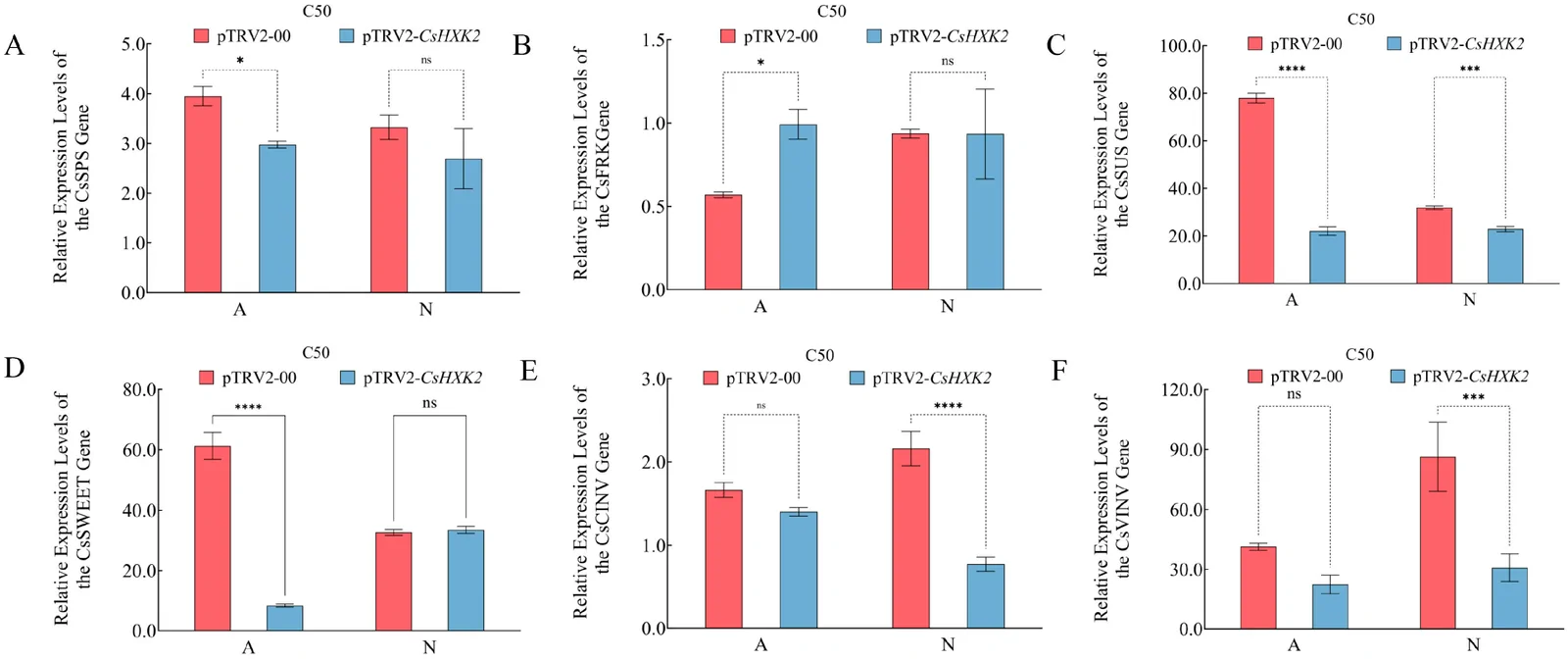
The effect of silencing on the expression levels of genes involved in sugar metabolism in the anthers and nectaries of C50. Note: (**A**): sucrose phosphate synthase, *CsSPS*; (**B**): fructokinase, *CsFRK*; (**C**): sucrose synthase, *CsSUS*; (**D**): transmembrane sugar transporter, CsSWEET; (**E**): alkaline invertase, *CsVINV*; (**F**): neutral invertase, *CsCINV*. Note: *, *p* < 0.05, ***, *p* < 0.001, ****, *p* < 0.0001 and ns, not significant (*p* > 0.05).

In the anthers of pTRV2-*CsHXK2* plants, *CsFRK* expression was significantly increased by 202.46% compared with the control. In contrast, the expression levels of *CsSPS*, *CsSUS*, *CsSWEET*, *CsCINV* and *CsVINV* were significantly decreased by 60.10%, 80.20%, 50.76%, 11.40%, and 74.49%, respectively. In the nectaries of pTRV2-*CsHXK2* plants, *CsFRK* expression was significantly increased by 38.27% compared with the control. Conversely, the expression levels of *CsSPS*, *CsSUS*, *CsSWEET*, *CsCINV*, and *CsVINV* were significantly decreased by 59.09%, 24.65%, 84.88%, 64.19%, and 57.04%, respectively. Furthermore, following *CsHXK2* silencing, the expression levels of *CsSPS*, *CsSUS*, and *CsSWEET* were higher in the anthers than in the nectaries, whereas *CsFRK*, *CsCINV*, and *CsVINV* exhibited the opposite expression pattern (Figure 8).

### 2.8. Expression Levels of Sugar Metabolism-Related Genes in Female Floral Organs

**Figure 9:**
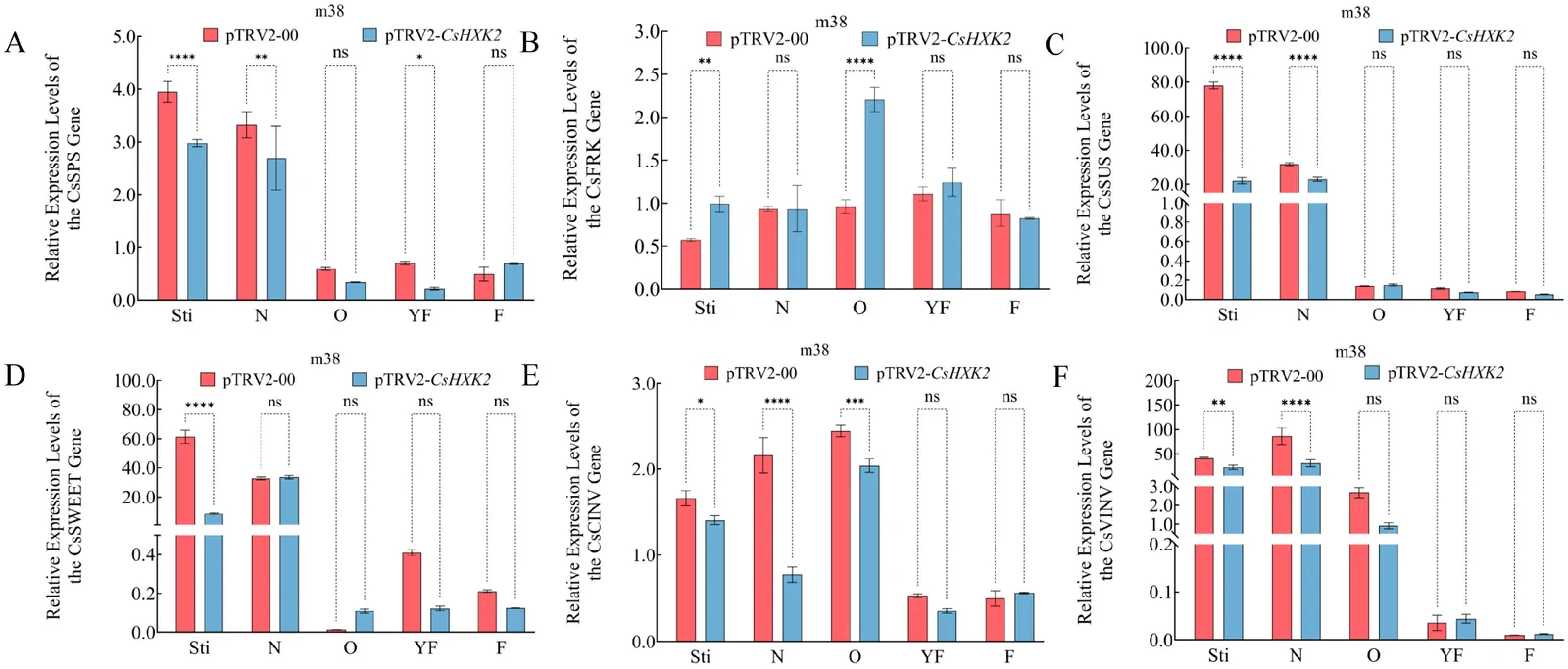
The effect of silencing on the expression levels of genes involved in sugar metabolism in the stigma, nectary, and ovary of m38. Note: (**A**): sucrose phosphate synthase, *CsSPS*; (**B**): fructokinase, *CsFRK*; (**C**): sucrose synthase, *CsSUS*; (**D**): transmembrane sugar transporter, *CsSWEET*; (**E**): alkaline invertase, *CsVINV*; (**F**): neutral invertase, *CsCINV*. *, *p* < 0.05, **, *p* < 0.01, ***, *p* < 0.001, ****, *p* < 0.0001 and ns, not significant (*p* > 0.05).

Following *CsHXK2* silencing, *CsSPS* expression levels in the stigmas, nectaries, and ovaries of m38 plants were significantly decreased by 43.69%, 41.69%, and 78.86%, respectively, compared with the control. The greatest reduction was observed in the ovaries, followed by the stigmas and nectaries (Figure 9).

In pTRV2-*CsHXK2* plants, *CsFRK* expression levels in the stigmas, nectaries, and ovaries. increased significantly by 212.81%, 53.78%, and 129.14%, respectively, relative to the control, with the highest expression observed in the ovaries, followed by the stigmas and then the nectaries.

In pTRV2-*CsHXK2* plants, *CsSUS* expression levels in the stigmas, nectaries and ovaries were significantly higher than the control by 183.32%, 56.78% and 86.10%, respectively, with the order being stigma > nectaries > ovary.

In plants with silenced *CsHXK2*, *CsSWEET* expression in the stigmas and ovaries was 70.41% and 758.60% higher than in the control, respectively. In contrast, expression in the nectaries decreased by 28.06%, with the following order of expression: stigma > nectaries > ovaries.

In pTRV2-*CsHXK2* plants, *CsVINV* and *CsCINV* expression levels in the nectaries were significantly reduced by 64.02% and 68.22%, respectively, compared with pTRV2-00 plants. Conversely, in pTRV2-*CsHXK2* plants, *CsVINV* and *CsCINV* expression levels were significantly increased (by 134.14% and 285.47%) and (by 22.81% and 41.21%), with nectaries > stigmas > ovaries.

## 3. Discussion

Plant hexokinase (HXK; EC 2.7.1.1) is a bifunctional enzyme that participates in both carbohydrate metabolism and sugar signaling, thereby contributing to plant growth, development, and responses to environmental stresses. In glycolysis, glucose-6-phosphate (G6P) is converted to fructose-6-phosphate (F6P) by glucose-6-phosphate isomerase and subsequently enters downstream reactions involved in energy metabolism. Alternatively, G6P can enter the pentose phosphate pathway, where glucose-6-phosphate dehydrogenase initiates its oxidation, ultimately contributing to the production of NADPH and ribose-5-phosphate required for cellular biosynthetic processes [20]. Furthermore, G6P can be transported into chloroplasts via glucose-6-phosphate transporters, where it contributes to starch biosynthesis [21].

The expression patterns of hexokinases are closely associated with plant developmental stages and tissue types. In oilseed rape, *BnHXK9* is highly expressed during early seed development, and RNA interference (RNAi) of *BnHXK9* results in delayed seed development [22]. In addition, *AtHKL3* and *OsHXK10* exhibit flower-specific expression [8,9]. In tomato, *SlHXK* genes are highly expressed in floral tissues, but individual family members exhibit distinct organ-specific expression patterns. *SlHXK1*, *SlHXK2*, *SlHXK3*, and *SlHXK5* are preferentially expressed in stamens, whereas *SlHXK4* is highly expressed in petals and *SlHXK6* is preferentially expressed in carpels [2]. In the present study, *CsHXK2* was expressed at markedly higher levels in the male and female flowers of ‘m43’ than in other tissues. Moreover, *CsHXK2* showed relatively high expression in the anthers and nectaries of the male line ‘C50’ and in the stigmas, nectaries, and ovaries of the female line ‘m38’. Collectively, these findings suggest that *HXK* family members may contribute to diverse developmental processes through spatially and temporally regulated expression patterns.

Plant hexokinases influence plant growth and development by modulating carbon metabolism and energy availability. Overexpression of *PbHXK1* in tomato significantly increased HXK activity and reduced sugar contents, while simultaneously inhibiting plant growth, as evidenced by shorter internodes and smaller leaves [23]. Transient overexpression of *ZjHXK5* and *ZjHXK6* in sugarcane, as well as their overexpression in tomato, significantly reduced total sugar content and the levels of individual soluble sugars. Conversely, transient silencing of *ZjHXK5* and *ZjHXK6* significantly increased sucrose and total sugar contents [24]. Consistent with these findings, silencing of *CsHXK2* in the present study significantly increased the contents of glucose, fructose, and sucrose in the anthers and nectaries of male flowers, as well as in the stigmas, nectaries, and ovaries of female flowers, compared with the control.

Hexokinase activity plays an important role in regulating carbon allocation and cellular energy homeostasis. For example, hexokinase activity in potato tubers is positively correlated with starch accumulation [25]; During embryo germination, *ZmHXK4* and *ZmHXK7* function in the mitochondria and cytoplasm, respectively, and contribute to the maintenance of cellular energy metabolism [26]. Under adverse environmental conditions, *HXK*-mediated regulation of sugar metabolism may contribute to stress acclimation. Under low-temperature conditions, *CsHXK1* expression is upregulated in tea plants, which may promote glucose-6-phosphate (G6P) production and support basal metabolism. Under salt stress, the *MdHXK1* protein phosphorylates the Na^+^/H^+^ exchanger *MdNHX1*, thereby contributing to cellular ion homeostasis [27]. Collectively, these metabolic regulatory mechanisms contribute to the maintenance of energy and metabolic homeostasis under adverse environmental conditions. In the present study, silencing of *CsHXK2* increased SPS and SS-II activities in the examined floral organs compared with the control, with more pronounced increases observed in the stigma and ovary than in the nectary and anther. Conversely, HXK, FRK, NI, SS-I, and AI activities were reduced in all examined floral organs following *CsHXK2* silencing, with generally greater reductions observed in female floral organs than in male floral organs. These results suggest that *CsHXK2* may exert a stronger regulatory effect on sugar-metabolic enzyme activities in female floral organs than in male floral organs. This differential response may be associated with differences in carbon demand and resource allocation between male and female flowers [28]. The distinct responses of male and female floral organs may also be related to sex-specific developmental programs in plants. In grapevine, the *VviPLATZ1* transcription factor has been implicated in the regulation of female flower morphology. The expression pattern of *VviPLATZ1* is closely associated with female floral organ development, particularly with the formation of reflexed stamens, a characteristic feature of female flowers [29]. In watermelon, the sex-determining gene *CitACS4* has been identified as a pleiotropic regulator of both floral and fruit development. Through its role in ethylene biosynthesis, *CitACS4* affects female flower development and is also associated with subsequent fruit formation [30].

Genes involved in sugar metabolism play important roles throughout plant growth and development by regulating sugar synthesis, transport, and allocation. The cucumber sugar transporter *CsSWEET5a* is localized to the plasma membrane and functions in hexose transport. *CsSWEET5a* is highly expressed in anthers and pollen from the microspore stage to the mature pollen stage and can partially complement the pollen defects of the Arabidopsis *atsweet8* mutant. These findings suggest that *CsSWEET5a* may contribute to hexose supply during pollen development [31]. Knockout of *OsFRK3* in rice reduces grain starch content, whereas *OsFRK3*-overexpressing lines exhibit increased starch accumulation [32]. In tomato, knockout of *SlCWINV* reduces fruit hexose content, whereas overexpression of *SlCWINV* promotes sugar accumulation [33]. The *NtNINV10*-GFP fusion protein in tobacco is localized to the plasma membrane, suggesting a potential role for *NtNINV10* in membrane-associated sucrose metabolism [34]. In the present study, silencing of *CsHXK2* significantly increased *CsFRK* expression in the anthers and nectaries of male flowers, whereas the expression levels of *CsSPS*, *CsSUS*, *CsSWEET*, *CsCINV*, and *CsVINV* were significantly decreased compared with the control. In female flowers, *CsFRK* and *CsSUS* expression levels in the stigmas, nectaries, and ovaries were significantly increased, whereas *CsSPS* expression was significantly decreased compared with the control. Moreover, *CsSWEET*, *CsVINV*, and *CsCINV* expression levels were increased in the stigmas and ovaries but decreased in the nectaries of *CsHXK2*-silenced female flowers compared with the control.

These results suggest that *CsHXK2* silencing alters sugar metabolic homeostasis and sugar signaling in both male and female cucumber flowers. *CsFRK* was consistently upregulated in multiple floral organs of both sexes, suggesting a potential compensatory response to reduced HXK-mediated hexose phosphorylation [35]. In contrast, *CsSPS* was consistently downregulated across the examined floral organs, suggesting that *CsHXK2* may be positively associated with the regulation of sucrose synthesis. Notably, the transcriptional responses differed between male and female flowers. In female floral organs, *CsSUS* was specifically upregulated, which may enhance sucrose cleavage and contribute to the maintenance of carbohydrate availability [36,37]. This tissue-specific response may reflect differences in sink strength and carbon demand among floral organs, with the stigma and ovary potentially receiving preferential carbon allocation to support fertilization and subsequent fruit development. Consistent with this possibility, *CsSWEET*, *CsCINV*, and *CsVINV* were upregulated in the stigma and ovary but downregulated in the nectary of female flowers. These contrasting expression patterns may contribute to tissue-specific differences in sugar transport, sucrose cleavage, and sugar accumulation [37]. In summary, these findings indicate that *CsHXK2* is involved in the regulation of sugar metabolism in both male and female cucumber flowers and may contribute to the redistribution of carbon resources among floral organs. The distinct transcriptional responses observed among reproductive organs further suggest that sugar metabolism may be differentially regulated according to organ-specific developmental demands, including pollen development in male flowers and fertilization and fruit development in female flowers.

Type B HXKs have been reported to function as both metabolic enzymes and sugar signaling regulators in various plant species. In cassava (*Manihot esculenta*), *MeHXK2* was shown to promote hexose phosphorylation and contribute to starch accumulation [38], whereas *CsHXK4* in tea plants was associated with glucose sensing and developmental regulation [3]. Similarly, *BnHXK9* in rapeseed was found to affect plant growth through modulation of sugar metabolism and signaling pathways [22]. These studies indicate that Type B HXKs exhibit conserved roles in maintaining carbon balance but may acquire species and tissue-specific functions. In the present study, silencing of *CsHXK2* resulted in altered expression of sugar metabolism-related genes and changes in the activities of key enzymes involved in sucrose and hexose metabolism, accompanied by increased soluble sugar accumulation in cucumber floral organs. Unlike previously characterized Type B HXKs that mainly function in storage organs, vegetative growth, or stress responses, our findings reveal a potential role of *CsHXK2* in regulating sugar homeostasis in cucumber reproductive organs. The differential responses between male and female flowers further suggest that *CsHXK2*-mediated sugar regulation may contribute to the distinct metabolic requirements during cucumber floral development.

Although VIGS-mediated silencing provided evidence supporting the involvement of *CsHXK2* in cucumber sugar metabolism and floral organ development, its broader effects on agronomic traits, such as plant growth, flower quality, and fruit yield, remain to be elucidated. Future studies using stable genetic materials and long-term phenotypic evaluations will be necessary to further clarify the contribution of *CsHXK2* to cucumber productivity and breeding improvement.

## 4. Materials and Methods

### 4.1. Plant Materials and Growth Conditions

The experiment was conducted in May 2025 in a plastic greenhouse at the Experimental Base of the Vegetable Research Institute, Gansu Academy of Agricultural Sciences, Lanzhou, Gansu Province, China (36°05′59″ N, 103°41′20″ E). The plant materials consisted of three cucumber genotypes: the bisexual-flowering germplasm ‘m43’, which produces comparable numbers of male and female flowers; the predominantly male line ‘C50’, which produces mainly male flowers with few female flowers; and the female line ‘m38’, which produces only female flowers. All three genotypes were developed by the Cucumber Research Group at the Vegetable Research Institute, Gansu Academy of Agricultural Sciences.

Seeds were surface-disinfected by soaking at 55 °C and then sown in 72-cell seedling trays. Seedlings were transplanted at the four-leaf-one-heart stage, with 30 plants established for each genotype at a spacing of 28 cm under a wide-furrow and narrow-bed cultivation system (70 cm/60 cm). Plants were grown under natural light conditions with an approximately 14 h light/10 h dark photoperiod. The greenhouse temperature was maintained at approximately 25–35 °C during the day and 10–20 °C at night, with a day/night temperature difference of approximately 10 °C. Plants were managed according to standard cucumber cultivation practices. Before transplanting, well-decomposed sheep manure was applied as basal fertilizer at a rate of 12 t ha^−1^. During the growth process, drip irrigation was applied based on the water and fertilizer requirements of the plants.

Samples of different tissues were collected at 20 days after the first female flower anthesis (20 DAF) for tissue-specific gene expression analysis. Roots, stems, leaves, male flowers, female flowers, and marketable fruits were collected from ‘m43’ for tissue-specific expression analysis. During the peak flowering period, petals, anthers, nectaries, and calyces were collected from male flowers of ‘C50’, whereas stigmas, nectaries, ovaries, petals, and calyces were collected from female flowers of ‘m38’. All samples were immediately frozen in liquid nitrogen and stored at −80 °C until further analysis.

### 4.2. Quantitative Real-Time PCR (qRT-PCR)

RNA extraction and cDNA synthesis were performed according to the method described by Mingming Dong et al. [39]. qRT-PCR was performed using SYBR GreenPro Taq HS Premix (AG11701, Aikrui, Changsha, China) on an L480 real-time PCR system (Bio-Rad, Hercules, CA, USA). The primers used for qRT-PCR are listed in Table 1. *CsActin* was used as the internal reference gene. Three biological replicates, each with three technical replicates, were performed for each sample, and relative gene expression levels were calculated using the 2^−ΔΔCt^ method [40].

### 4.3. Construction and Transformation of the Subcellular Localization Vector

Using cDNA derived from the nectary of ‘m38’ as a template, the coding sequence of *CsHXK2* was amplified for construction of a subcellular localization vector. The amplified fragment was ligated into the pCAMBIA1300-GFP vector using the *BamH*I and *Sal*I restriction sites. The recombinant plasmid was subsequently introduced into competent *Escherichia coli* cells. Individual colonies were selected and screened by colony PCR, and positive clones were subsequently confirmed by sequencing at Qingke Biotechnology Co., Ltd. (Xi’an, China). After sequence verification, the recombinant plasmid was extracted and introduced into competent *Agrobacterium tumefaciens* GV3101 cells using the freeze–thaw method.

An infiltration buffer containing 10 mM MES, 10 mM MgCl_2_, and 200 μM AS (pH 5.7) was prepared. The Agrobacterium suspension was resuspended in the buffer and incubated at 28 °C with gentle shaking in the dark for 3 h. Healthy 3–4-week-old tobacco plants were selected for transient expression assays. A small puncture was made on the abaxial surface of each leaf with a needle, and approximately 1 mL of the Agrobacterium infiltration suspension was infiltrated into the leaf using a needleless syringe. Following infiltration, the tobacco plants were maintained in the dark for 48 h. GFP fluorescence in the infiltrated leaves was subsequently observed using a confocal laser scanning microscope (LSM 800, Zeiss, Oberkochen, BW, Germany) with an excitation wavelength of 488 nm and an emission wavelength of 510 nm.

### 4.4. Virus-Induced Gene Silencing (VIGS)

In this study, the VIGS (virus-induced gene silencing) system was established using the dual-vector system pTRV1 and pTRV2, based on the tobacco crispvirus. A recombinant TRV2-*CsHXK2* vector containing *EcoR*I and *BamH*I restriction sites was constructed by homologous recombination. After sequence verification, the recombinant plasmid was introduced into competent *Agrobacterium tumefaciens* GV3101 cells. Agrobacterium suspensions carrying TRV1 were mixed separately with suspensions carrying either TRV2 or TRV2-*CsHXK2* at a 1:1 (*v*/*v*) ratio and infiltrated into the cotyledons of C50 and m38 seedlings. Ten seedlings were used for each treatment. Following infiltration, the treated cucumber seedlings were maintained in darkness at 18 °C for 3 d. The seedlings were then transferred to standard growth conditions with a day/night temperature of 22/18 °C, a light intensity of 20,000 lx, and a photoperiod of 16 h light/8 h dark. The expression levels of *CsHXK2* were quantified by qRT-PCR in anthers and nectaries of male flowers and in stigmas, nectaries, and ovaries of female flowers from plants inoculated with TRV2 (empty-vector control) or TRV2-*CsHXK2*.

### 4.5. Assessment of Gene Silencing Efficiency and Sugar Contents in Silenced Plants

During the peak flowering period, anthers and nectaries from male flowers and stigmas, nectaries, and ovaries from female flowers were collected from pTRV2-00 and pTRV2-*CsHXK2* plants. The samples were immediately frozen in liquid nitrogen and stored in an ultra-low-temperature freezer until further analysis. The expression levels of *CsHXK2* were quantified by qRT-PCR, and gene silencing efficiency was calculated accordingly.

Following the method described by He Yajuan et al. [21], the concentrations of sucrose, glucose, and fructose in the collected floral tissues were quantified using a Waters ACQUITY Arc high-performance liquid chromatography (HPLC) system (Waters, Milford, MA, USA). The calibration curves for glucose, fructose, and sucrose were y = 48,660x + 60,085 (R^2^ = 0.9995), y = 8866.1x + 107,184 (R^2^ = 0.9625), and y = 21,139x + 1638.1 (R^2^ = 0.9995), respectively. Although the R^2^ value for fructose was slightly lower than those of the other sugars, it still met the requirements for quantitative analysis and ensured reliable determination of fructose content in cucumber samples.

### 4.6. Assessment of Enzyme Activities and Expression Levels of Sugar Metabolism-Related Genes Following CsHXK2 Silencing

Approximately 0.1 g of anther and nectary tissues from male flowers, as well as stigma, nectary, and ovary tissues from female flowers, were separately collected. Each sample was homogenized with 500 μL of extraction buffer on ice until a uniform suspension was obtained. The activities of hexokinase (HXK; Cat No. CB10058-Pt), sucrose-phosphate synthase (SPS; Cat No. CB10137-Pt), phosphofructokinase (PFK; Cat No. CB10068-Pt), sucrose synthase I (SS-I/SS-C; Cat No. CB10802-Pt), sucrose synthase II (SS-II/SS-S; Cat No. CB10803-Pt), cytosolic neutral invertase (NI; Cat No. CB10283-Pt), and soluble acid invertase (AI; Cat No. CB10282-Pt) were measured using plant-specific ELISA kits (Keaibo Biotechnology, Shanghai, China; stored at 2–8 °C). Briefly, 50 μL of standards or sample extracts was added to pre-coated microplate wells, followed by the addition of 100 μL of HRP-labeled detection antibody. The plates were incubated for 60 min at 37 °C. After washing the wells five times, 50 μL of substrate A and 50 μL of substrate B were added, followed by incubation for 15 min at 37 °C in the dark. The reaction was terminated by adding stop solution, and the optical density (OD) values were measured at 450 nm within 15 min. Enzyme activities were calculated based on the corresponding standard curves. All procedures were performed according to the manufacturer’s instructions.

qRT-PCR was used to quantify the expression levels of the sugar metabolism-related genes *CsSUS*, *CsSPS*, *CsFRK*, *CsSWEET*, *CsCINV*, and *CsVINV* in pTRV2-00 control plants and pTRV2-*CsHXK2* silenced plants.

### 4.7. Statistical Analysis

Data analysis and processing were performed using Excel 2024, whilst GraphPad Prism (10.1.2) was used to conduct significance tests, calculate standard errors and generate graphs. A one-way analysis of variance (ANOVA) was employed to compare differences between groups, with Šídák’s multiple comparison test used for pairwise comparisons; differences were considered statistically significant at *p* < 0.05.

## 5. Conclusions

Silencing of *CsHXK2* promoted sugar accumulation in cucumber floral organs and altered the activities of key sugar metabolic enzymes, as well as the expression of genes involved in sugar metabolism. These findings suggest that *CsHXK2* plays an important role in maintaining sugar homeostasis in cucumber floral organs. Male and female floral organs exhibited distinct responses to *CsHXK2*-mediated changes in sugar metabolism. These differences may influence cucumber floral development by modulating carbon allocation and sugar signaling. Future research should focus on elucidating the physiological and genetic mechanisms of *CsHXK2*-mediated sugar regulation and exploring its potential application in cucumber breeding to improve floral traits and yield performance.

## Figures and Tables

**Table 1:** Primer information used in this study.

Primer Name	Primer Sequence (5′−3′)	Usage
CsActin-F	GCCCTCCCTCATGCCATTCT	Fluorescence quantification
CsActin-R	TCGGCAGTGGTGGTGAACAT	
CsHXK2-F	ATGAAGAAGGTTGTTGTTGGAACC	
CsHXK2-R	GGACTCCTCTACCCCAAGATACTG	
CsSUS-F	ATCGTTGGGTTTCTCGTT	
CsSUS-R	TTGGGTTACTCCTAATCTGC	
CsSPS-F	CAAGTCATTATCCAGGTAGA	
CsSPS-R	CCAAGGTTGAGCCAGTTT	
CsCINV-F	ATACCGTTGCTGCTGATT	
CsCINV-R	CATTCCCTTCTGACACTC	
CsVINV-F	GGAATGTGGGAATGTCTA	
CsVINV-R	CTTGGGATTATCAGGAAC	
CsFRK-F	AGCTTTCATTGGCAAGGTAGGTG	
CsFRK-R	CATTCGGGTCAAACCGCACT	
CsSWEET-F	GCCGCATCCCAGTGAAGGA	
CsSWEET-R	TGACGGGCACAGGGTTTAGTTC	
GFP-CsHXK2-F	gagctcggtacccggggatccATGAAGAAGGTTGTTGTGGAACC	Subcellular localisation
GFP-CsHXK2-R	cttgctcaccatggtgtcgacGGACTCCTCTACCCCAAGATACTG	
TRV-CsHXK2-F	gtgagtaaggttaccgaattcATGAAGAAGGTTGTTGTTGGAACC	Gene silencing
TRV-CsHXK2-R	cgtgagctcggtaccggatccAGCAACACGATCATCCTTTCCT	

## Data Availability

The original contributions presented in this study are included in the article. Further inquiries can be directed to the corresponding authors.
